# Metal Homeostasis and Exposure in Distinct Phenotypic Subtypes of Insulin Resistance among Children with Obesity

**DOI:** 10.3390/nu15102347

**Published:** 2023-05-17

**Authors:** Álvaro González-Domínguez, María Millán-Martínez, Jesús Domínguez-Riscart, Alfonso María Lechuga-Sancho, Raúl González-Domínguez

**Affiliations:** 1 Instituto de Investigación e Innovación Biomédica de Cádiz (INiBICA), Hospital Universitario Puerta del Mar, Universidad de Cádiz, 11009 Cádiz, Spain; 2 Associate Unit CSIC-University of Huelva “Atmospheric Pollution”, Center for Research in Sustainable Chemistry-CIQSO, University of Huelva, 21071 Huelva, Spain; 3 Department of Chemistry, Faculty of Experimental Sciences, University of Huelva, 21071 Huelva, Spain; 4 Unidad de Endocrinología Pediátrica y Diabetes, Servicio de Pediatría, Hospital Universitario Puerta del Mar, 11009 Cádiz, Spain; 5 Departamento Materno Infantil y Radiología, Facultad de Medicina, Universidad de Cádiz, 11009 Cádiz, Spain

**Keywords:** childhood obesity, insulin resistance, trace elements, insulin secretion profile, delayed hyperinsulinemia, oral glucose tolerance test

## Abstract

Background: Trace elements and heavy metals have proven pivotal roles in childhood obesity and insulin resistance. However, growing evidence suggests that insulin resistance could encompass distinct phenotypic subtypes. Methods: Herein, we performed a comprehensive metallomics characterization of plasma samples from children and adolescents with obesity and concomitant insulin resistance, who were stratified as early (N = 17, 11.4 ± 2.4 years), middle (N = 16, 11.8 ± 1.9 years), and late (N = 33, 11.7 ± 2.0 years) responders according to the insulin secretion profile in response to an oral glucose tolerance test. To this end, we employed a high-throughput method aimed at determining the biodistribution of various essential and toxic elements by analyzing total metal contents, metal-containing proteins, and labile metal species. Results: Compared with the early responders, participants with delayed glucose-induced hyperinsulinemia showed a worsened insulin resistance (HOMA-IR, 4.5 vs. 3.8) and lipid profile (total cholesterol, 160 vs. 144 mg/dL; LDL-cholesterol, 99 vs. 82 mg/dL), which in turn was accompanied by sharpened disturbances in the levels of plasmatic proteins containing chromium (4.8 vs. 5.1 µg/L), cobalt (0.79 vs. 1.2 µg/L), lead (0.021 vs. 0.025 µg/L), and arsenic (0.077 vs. 0.17 µg/L). A correlation analysis demonstrated a close inter-relationship among these multielemental perturbations and the characteristic metabolic complications occurring in childhood obesity, namely impaired insulin-mediated metabolism of carbohydrates and lipids. Conclusions: These findings highlight the crucial involvement that altered metal homeostasis and exposure may have in regulating insulin signaling, glucose metabolism, and dyslipidemia in childhood obesity.

## 1. Introduction

Obesity is currently the most prevalent noncommunicable disorder among children and adolescents from developed countries. This disorder is often accompanied by a multitude of metabolic complications, such as insulin resistance (IR), impaired fasting glucose, impaired glucose tolerance, and dyslipidemia, which altogether increase the risk of developing type 2 diabetes and cardiovascular events [1]. Among them, IR has been identified as a precursor in disturbed carbohydrate metabolism in childhood obesity, whereas glycemic alterations frequently occur at older ages [1]. However, growing evidence suggests that IR could be a heterogeneous disorder comprising distinct phenotypic subtypes, which in turn are differentially associated with the typical metabolically unhealthy sequelae underlying obesity. In this respect, the pattern of insulin secretion during an oral glucose tolerance test (OGTT) has been proposed as a reliable indicator of the severity of insulin-related metabolic defects [2]. In response to glucose stimulation, pancreatic β-cells secrete insulin in a biphasic manner. The first phase is characterized by a fast and transient increase in blood insulin concentrations within 5–10 min, whereas the late phase occurs over the course of hours and persists as long as the circulating glucose levels remain elevated [3]. A late postprandial insulinemic peak has been associated with early β-cell dysfunction and decreased insulin sensitivity in adults, as well as with higher odds of subsequent type 2 diabetes [2]. Similar results have recently been reported in children with obesity, with lower insulin sensitivity and worse metabolic profiles being observed among subjects with delayed insulin secretion in response to an OGTT [4]. Furthermore, late glucose-induced hyperinsulinemia seems to be closely linked to dyslipidemia factors [5]. Altogether, the assessment of the insulinemic profile could be a powerful tool in the context of precision medicine with the aim of investigating within-group differences driven by insulin-related interindividual variability factors.

In this respect, it has been demonstrated that numerous metal and metalloid elements play a pivotal role in regulating insulin homeostasis and, consequently, in influencing carbohydrate and lipid metabolism. Various trace essential metals, including zinc, chromium, vanadium, molybdenum, and cobalt, have been found to be directly involved in the synthesis, storage, and activity of insulin [6]. Because of their antioxidant properties, other elements (e.g., selenium and manganese) are well known to mitigate insulin defects caused by oxidative damage in pancreatic β-cells. Obesity and comorbidities have also been linked to important perturbations in iron metabolism, which are closely related to inflammatory processes and exacerbated oxidative stress [7]. On the other hand, toxic heavy metals, such as cadmium, mercury, and lead, may impair glycemic control by triggering chronic inflammation, oxidative stress, and endocrine disruption [8]. Accordingly, considerable efforts have been made in recent years to investigate the potential involvement of metals in the pathogenesis of obesity and related comorbidities. Observational studies have reported that childhood obesity is characterized by an increased blood content of copper together with decreased iron, zinc, and selenium levels, but the results are contradictory for other minor elements [9,10,11,12]. Furthermore, recent evidence suggests that sex and pubertal status could influence obesity-related metal alterations [13,14]. However, the significance of IR subtypes in these metal disturbances remains unexplored.

In this work, we aimed to unveil the relationship between circulating metal species and the insulin secretion profile in children and adolescents with obesity and IR, who were stratified into three subgroups based on their individual response to an OGTT: subjects with an early insulin peak (t = 30 min), subjects with a middle insulin peak (t = 60 min), and subjects with a late insulin peak (t ≥ 90 min). For this purpose, fasting plasma samples were subjected to a multielemental analysis using a state-of-the-art metallomics method that enables quantifying the total metal contents, as well as to fractionate metal species for characterizing the metalloproteome and the labile metal pool [15,16].

## 2. Materials and Methods

### 2.1. Study Design

The study population consisted of children and adolescents with obesity and concomitant IR enrolled at Hospital Universitario Puerta del Mar (Cádiz, Spain). Obesity was defined as presenting a body mass index (BMI) over two standard deviations above the mean of the reference population, adjusted for sex and age [17]. Children with other known chronic systemic diseases or suffering of acute infectious processes were not eligible for the study. Participants underwent an OGTT to diagnose IR using the criteria described elsewhere [12,18]. In turn, the measurement of blood insulin along the OGTT curve was employed to stratify the population into three subgroups based on the insulin secretion profile, as previously reported [2,4]: subjects with early insulin peak (t = 30 min, N = 17), subjects with middle insulin peak (t = 60 min, N = 16), and subjects with late insulin peak (t ≥ 90 min, N = 33). The participants in the different study groups had comparable Tanner stages (ca. 30% prepuberal children), and none of them were active smokers neither in the present nor the past. From all subjects, blood samples were collected in the morning after overnight fasting, as well as at the different time points along the OGTT (i.e., 30, 60, 90, and 120 min), using BD Vacutainer EDTA tubes and Advance vacuum system. Blood tubes were centrifuged at 1500× *g* for 10 min at 4 °C to separate the plasma, which was aliquoted and stored at −80 °C until analysis. The study was performed in accordance with the principles contained in the Declaration of Helsinki. The Ethical Committee of Hospital Universitario Puerta del Mar (Cádiz, Spain) approved the study protocol (Ref. PI22/01899), and all participants and/or legal guardians provided written informed consent.

### 2.2. Measurement of Anthropometric and Biochemical Variables

Anthropometric variables (i.e., weight, height, BMI, and waist circumference) were evaluated by pediatric endocrinologists. Glucose and insulin concentrations along the OGTT curve (i.e., 0, 30, 60, 90, and 120 min), as well as fasting levels of glycated hemoglobin (HbA1c) and lipid profile (i.e., total cholesterol, TC; high-density lipoprotein cholesterol, HDL-C; low-density lipoprotein cholesterol, LDL-C; triglycerides, TGs), were measured using an Alinity automatic analyzer (Abbot, Madrid, Spain). The homeostasis model assessment of insulin resistance (HOMA-IR), the whole-body insulin sensitivity index (WBISI), the area under the curve for glucose (AUC_Glc_), the area under the curve for insulin (AUC_Ins_), and the Castelli risk index-I (CRI-I) were calculated by applying the following Formulas (1)–(5):

HOMA-IR = (Glc_0_ × Ins_0_) × 0.055/22.5 (1)

WBISI = 10,000/(Glc_0_ × Ins_0_ × Glc_mean_ × Ins_mean_)^1/2^ (2)

AUC_Glc_ = 0.25 × Glc_0_ + 0.5 × Glc_30_ + 0.5 × Glc_60_ + 0.5 × Glc_90_ + 0.25 × Glc_120_ (3)

AUCIns = 0.25 × Ins0 + 0.5 × Ins30 + 0.5 × Ins60 + 0.5 × Ins90 + 0.25 × Ins120(4)

CRI-I = TC/HDL-C(5)

### 2.3. Multielemental Analysis

Total metal contents were determined by simple dilution of plasma samples, whereas protein precipitation under nondenaturing conditions was applied for size fractionation of high-molecular-mass (i.e., HMM) and low-molecular-mass (i.e., LMM) metal species, following the methodology described by González-Domínguez et al. [16]. Then, multielemental analyses were performed in an inductively coupled plasma mass spectrometer, using the method optimized elsewhere [16].

### 2.4. Dietary Assessment and Association with Arsenic Exposure

Dietary habits of the study participants were assessed using the KIDMED questionnaire [19]. A metal–diet association study was performed among elements showing differential content among the study groups and plausible food sources contained in the KIDMED questionnaire. Thus, we particularly focused on the fifth item of this questionnaire regarding the consumption of seafood products. The chi-square test was applied to look for differences among the study groups, and point-biserial correlation was employed to evaluate the association between dietary data and plasma arsenic concentrations.

### 2.5. Statistical Analysis

Analysis of variance (ANOVA) followed by the Fisher LSD post hoc test was applied to compare clinical and biochemical characteristics among study groups. The preprocessing and statistical analysis of metallomics data were performed using the MetaboAnalyst 5.0 web tool (https://www.metaboanalyst.ca/) (accessed on 1 February 2023) according to the pipeline described elsewhere [12], comprising missing value imputation, data scaling and transformation, and further univariate analysis by ANOVA with Fisher LSD post hoc test and Benjamini–Hochberg false discovery rate (FDR) correction. Furthermore, linear models with covariate adjustment were complementarily employed to control for the influence of potential confounding factors (i.e., Tanner stage). Finally, Pearson’s correlations were computed between significant metals and other biochemical variables.

## 3. Results

The participants were on average 11.6 years old, and 57.1% were male. Demographic and anthropometric characteristics were similar among the three study groups (Table 1). In addition to the expected differences in blood insulin, participants with delayed hyperinsulinemia also had sustained elevated blood glucose concentrations at the end of the OGTT curve (i.e., 90 and 120 min) and higher HOMA-IR scores. However, the rest of the parameters relating to insulin and glucose control (e.g., WBISI and AUCs for insulin and glucose) did not differ among the study groups. Regarding lipid metabolism, subjects with early glucose-induced hyperinsulinemia displayed a healthier lipid profile, with lower TC and LDL-C levels (Table 1), in line with other studies [4,5]. The consumption of seafood products was significantly lower in participants with delayed insulin response.

A multielemental analysis of plasma samples enabled us to comprehensively investigate the association between the insulin secretion profile and changes in circulating metal species among children and adolescents with obesity and IR (Table 2). The total metal contents measured in this study covered a wide range of concentrations, from ng/L for minor toxic heavy metals (e.g., cadmium and lead) up to mg/L for major essential elements (e.g., copper), in agreement with previous data [20]. Most of these elements were majorly biodistributed within the HMM fraction in the form of metalloproteins, whereas the levels of labile species were much lower or even negligible, as reported elsewhere [21,22]. The statistical comparison of the study groups revealed significant differences in various HMM metal species, but not in their total or LMM contents (Table 2). Delayed hyperinsulinemia was characterized by lower arsenic, chromium, cobalt, and lead metalloproteins compared to subjects with early and middle insulin peaks. The application of linear modeling with covariate adjustment demonstrated that the Tanner stage did not have a significant impact on the results (As, raw *p*-value 0.0015, adjusted *p*-value 0.038; Co, raw *p*-value 0.0015, adjusted *p*-value 0.038; Cr raw *p*-value 0.038, adjusted *p*-value 0.045; and Pb, raw *p*-value 0.038, adjusted *p*-value 0.045). The same trend was observed for total (As, Cr, and Pb) and LMM (As and Cr) fractions without reaching statistical significance. The levels of the rest of the elements under investigation did not differ among the study groups.

**Figure 1:**
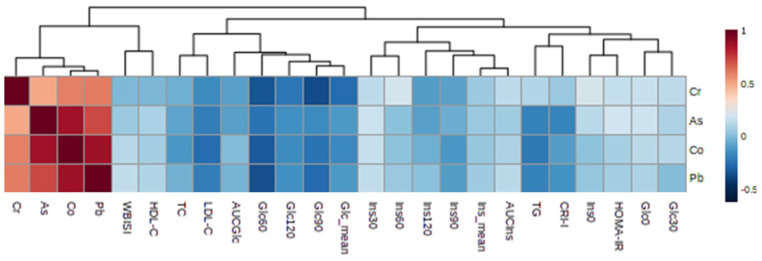
Pearson’s correlation matrix between significant metals and biochemical variables.

Finally, a correlation analysis demonstrated a close inter-relationship between the multielemental alterations described above and the characteristic metabolic complications behind childhood obesity and IR, including impaired insulin-mediated carbohydrate metabolism and dyslipidemia (Figure 1). Plasma glucose concentrations along the OGTT curve showed a consistent negative association with the HMM species of arsenic (Glc_60_, r = −0.23), chromium (Glc_60_, r = −0.33; Glc_90_, r = −0.37; Glc_120_, r = −0.22; Glc_mean_, r = −0.27), cobalt (Glc_60_, r = −0.31; Glc_90_, r = −0.25), and lead (Glc_60_, r = −0.34; Glc_90_, r = −0.26). Regarding lipid parameters, cobalt in the HMM fraction was negatively correlated with LDL-C (r = −0.27) and TG (r = −0.22) levels, whereas Pb-containing metalloproteins negatively correlated with LDL-C (r = −0.21). Interestingly, arsenic in the HMM fraction was also positively correlated with the frequency of seafood consumption (r = 0.25), as evaluated through the KIDMED questionnaire.

## 4. Discussion

To the best of our knowledge, this is the first study that has evaluated the interplay between circulating metals and the glucose-induced insulin secretion pattern among children and adolescents with obesity and concomitant IR. Our results suggest that delayed hyperinsulinemia in response to an OGTT is associated with lower plasmatic contents of arsenic, chromium, cobalt, and lead. These metallomics differences were particularly relevant when analyzing the HMM metal fraction, which emphasizes the added value of the size fractionation method employed here over simply determining total metal contents, as commonly reported in the literature. Interestingly, a correlation analysis evidenced a consistent association between these significant metal species and biochemical variables related to carbohydrate and lipid metabolism, thus highlighting the pivotal role that metals may play in the characteristic metabolic complications that frequently underly obesity and related comorbidities.

One of the most remarkable findings of this study was the significant reduction in the plasma contents of metalloproteins containing chromium and cobalt among subjects with late insulin peak (Table 2), which in turn showed a strong negative correlation with several markers of hyperglycemia and dyslipidemia (the latter only for HMM-Co species, Figure 1). This concurs with available scientific evidence that supports the pivotal involvement of these metals in regulating insulin homeostasis and, consequently, in controlling glucose and lipid metabolism. On the one hand, it has been reported that chromium participates in a myriad of biological functions that result in improved insulin signaling and glucose control (e.g., tyrosine kinase receptor activation and glucose transporter 4 translocation) and in a healthier lipid profile (e.g., enhanced β-oxidation and inhibited cholesterol synthesis) [23]. Similarly, cobalt has also been associated with improved glucose tolerance, glucagon signaling suppression, and amelioration of dyslipidemia factors [24,25]. In this respect, some observational studies have linked childhood obesity to lower blood levels of chromium and cobalt [11,12,26]. Accordingly, the results found herein reinforce the hypothesis that perturbations in these essential trace elements could be behind the insulin-mediated pathogenic events occurring in obesity, which are expected to be exacerbated among subjects with a delayed insulin response against a glycemic challenge.

Several studies have previously reported an inverse association between lead levels in different biological matrices, such as blood [27,28], urine [27,29], and bone [30], and the body weight and adiposity of children and adolescents. Although the evidence is not conclusive, various mechanisms have been hypothesized to account for this association based on data from animal models. First, it has been demonstrated that lead exposure may induce appetite suppression via downregulating the food satiety signals, thereby provoking a reduction in food intake and, consequently, in body weight [31,32]. More recently, various authors have reported that appetite suppression triggered by lead could be mediated by disturbances in the hypothalamic dopaminergic system, which regulates reward-seeking behaviors [33,34]. Moreover, this heavy metal can also disrupt the hypothalamic–pituitary–adrenal (HPA) axis through an interaction with glucocorticoids (e.g., cortisol), hormones that are responsible for controlling food intake and energy expenditure [35]. Within this tangled meshwork, it should also be noted that lead may affect glucose metabolism and insulin signaling pathways [8], which could account for the differences observed in the present study between the distinct IR subtypes (Table 2). Additionally, in accordance with the results from our correlation analyses (Figure 1), low blood lead has previously been associated with higher odds of dyslipidemia, plausibly because of the ability of this metal to interfere with cholesterol metabolism [27,30]. However, we would like to caution here that these findings do not imply that lead should be considered a protective element against obesity and IR, especially considering its toxic properties as an endocrine disruptor.

Finally, arsenic-containing HMM species were also found to be decreased among subjects with late hyperinsulinemia, and the same but nonsignificant behavior was found for total and LMM contents (Table 2). In this respect, it is important to highlight that previous human epidemiologic studies on the association between this metalloid element and obesity are rather inconsistent [36]. As arsenic is majorly incorporated into the organism though diet and other environmental sources, these discrepancies could be mainly allocated to differences in exposure levels between the populations under study [37]. This is of relevance considering the hormetic characteristics of this trace element, which is toxic at high doses, whereas low levels seem to be essential for various biological processes, such as methionine metabolism, DNA synthesis, and cell repair mechanisms [38]. Herein, the evaluation of the food intake questionnaires showed that the frequency of consumption of seafood products (i.e., the main source of arsenic) was significantly lower in participants with delayed insulin response and was in turn positively correlated with blood arsenic levels. This concurs with recent data evidencing a direct association between the adherence to a Mediterranean diet and plasma arsenic levels among children with obesity [39]. Thus, these results support the rationale that nutritional deficiencies could be, at least in part, responsible for the inverse association between arsenic and IR severity among children and adolescents with obesity. Alternatively, another plausible explanation for this observation could be the occurrence of disturbances in the metabolism (e.g., methylation capacity) and excretion of arsenic species. In this sense, it has been reported that decreased urinary arsenic levels in obesity could be an indicator of an abnormal biodistribution toward its storage and accumulation in adipose tissue [40], which subsequently may trigger obesity-related IR [41]. Altogether, it is unclear whether our findings truly reflect lower exposure or, instead, are related to metabolic perturbations that alter arsenic metabolism and tissue sequestration.

The major strength of this work was the investigation, for the first time, of the role that insulin-related interindividual variability factors may have in the association between childhood obesity and metal homeostasis/exposure. This was possible thanks to the study of a well-characterized population comprising children and adolescents with obesity, who underwent an OGTT with the aim of assessing their capacity of properly secreting insulin in response to a glycemic challenge (i.e., early vs. late responders). We also leveraged a state-of-the-art metallomics method, which enables simultaneously analyzing total metal contents, metal-containing proteins, and labile metal species, to perform a deep characterization of the biodistribution of trace elements and heavy metals in blood. However, future studies are required to better characterize this interplay among metals, obesity, and IR, as well as to elucidate the exact mechanisms underlying these associations. In this respect, the main limitations of this study were the relatively small sample size and the lack of an independent cohort for validation purposes. This is particularly relevant considering the inherent lowering in statistical power that stratification according to the insulin secretion profile in response to an OGTT entails. Furthermore, the complementary analysis of other biological matrices could provide a more comprehensive overview of the alterations occurring in metal homeostasis. This is of special relevance taking into consideration that part of the findings reported here were related to metals that are primarily associated with exposure rather than endogenous metabolism (i.e., lead and arsenic), which would make urine a biological sample of great interest.

## 5. Conclusions

In summary, we investigated for the first time the differences in circulating metal species among distinct IR phenotypic subtypes among children and adolescents with obesity, who were stratified according to the insulin secretion pattern in response to an OGTT. We found that delayed glucose-induced hyperinsulinemia was associated with lower levels of chromium, cobalt, lead, and arsenic, particularly at the metalloproteome level. These results highlight the pivotal role that trace elements and exposure to heavy metals may have in regulating insulin signaling and, consequently, in controlling glucose and lipid metabolism. Altogether, this study lays the foundation for understanding the crucial importance of insulin-related interindividual variability factors in metal homeostasis and emphasizes the need for further investigations to develop more effective preventive, diagnostic, and treatment approaches in youth obesity.

## Figures and Tables

**Table 1:** Characteristics of the study population, expressed as mean ± standard deviation (except for sex, expressed as percentage). NS, nonsignificant.

	Early Peak	Middle Peak	Late Peak	*p*-Value
Demographic, Anthropometric, and Dietary Variables				
N	17	16	33	NS
Age (years)	11.4 ± 2.4	11.8 ± 1.9	11.7 ± 2.0	NS
Sex (% male)	60.6	56.3	54.5	NS
Weight (kg)	72.1 ± 22.8	72.1 ± 19.8	72.3 ± 16.8	NS
Body mass index (BMI, kg/m^2^)	31.0 ± 5.9	29.2 ± 5.4	30.6 ± 5.6	NS
Waist circumference (WC, cm)	98.3 ± 12.8	98.5 ± 14.7	99.9 ± 11.7	NS
Frequency of seafood consumption (at least 2–3 per week, %)	100.0 ^a^	66.7 ^b^	60.0 ^b^	4.8 × 10^−2^
Carbohydrate metabolism				
Fasting glucose (Glc_0_, mg/dL)	85.8 ± 10.9	85.4 ± 7.8	83.8 ± 8.1	NS
Postprandial glucose, t = 30 min (Glc_30_, mg/dL)	150.0 ± 21.4	141.3 ± 31.0	133.3 ± 22.0	NS
Postprandial glucose, t = 60 min (Glc_60_, mg/dL)	123.4 ± 19.1	138.4 ± 27.6	142.9 ± 33.2	NS
Postprandial glucose, t = 90 min (Glc_90_, mg/dL)	116.2 ± 11.8 ^a^	111.6 ± 28.2 ^a^	138.9 ± 31.2 ^b^	4.2 × 10^−3^
Postprandial glucose, t = 120 min (Glc_120_, mg/dL)	113.6 ± 13.7 ^a^	118.3 ± 28.5 ^a^	134.8 ± 29.3 ^b^	1.5 × 10^−2^
Mean glucose (Glc_mean_, mg/dL)	119.9 ± 19.1	119.8 ± 21.2	131.2 ± 21.4	NS
Area under the curve for glucose (AUC_Glc_, mg·h/dL)	202.9 ± 59.0	200.2 ± 52.6	220.9 ± 51.7	NS
Fasting insulin (Ins_0_, µU/mL)	18.6 ± 4.3	19.2 ± 6.1	22.1 ± 11.7	NS
Postprandial insulin, t = 30 min (Ins_30_, µU/mL)	242.1 ± 135.4 ^a^	114.1 ± 56.1 ^b^	122.0 ± 62.3 ^b^	9.8 × 10^−6^
Postprandial insulin, t = 60 min (Ins_60_, µU/mL)	168.7 ± 96.9 ^a^	213.7 ± 69.2 ^b^	135.1 ± 73.5 ^c^	4.8 × 10^−3^
Postprandial insulin, t = 90 min (Ins_90_, µU/mL)	138.4 ± 75.7	141.8 ± 70.9	177.9 ± 111.7	NS
Postprandial insulin, t = 120 min (Ins_120_, µU/mL)	126.3 ± 70.1 ^a^	154.1 ± 93.8 ^ab^	176.1 ± 102.6 ^b^	4.8 × 10^−2^
Mean insulin (Ins_mean_, µU/mL)	149.5 ± 86.5	128.9 ± 48.2	130.1 ± 57.2	NS
Area under the curve for insulin (AUC_Ins_, µU·h/mL)	327.5 ± 191.1	274.6 ± 98.6	266.9 ± 123.8	NS
HOMA-IR	3.8 ± 1.2 ^a^	4.0 ± 1.3 ^a^	4.5 ± 1.6 ^b^	4.8 × 10^−2^
WBISI	0.20 ± 0.079	0.25 ± 0.13	0.22 ± 0.11	NS
Glycated hemoglobin (HbA1c, %)	5.3 ± 0.3	5.3 ± 0.3	5.2 ± 0.3	NS
Lipid metabolism				
Total cholesterol (TC, mg/dL)	144 ± 23 ^a^	164 ± 35 ^b^	160 ± 31 ^b^	3.7 × 10^−2^
Low-density lipoprotein cholesterol (LDL-C, mg/dL)	82 ± 18 ^a^	97 ± 28 ^b^	99 ± 29 ^b^	1.3 × 10^−2^
High-density lipoprotein cholesterol (HDL-C, mg/dL)	42 ± 8	45 ± 9	47 ± 26	NS
Castelli risk index-I (CRI-I)	3.4 ± 0.9	3.5 ± 0.8	3.9 ± 0.8	NS
Triglycerides (TGs, mg/dL)	100 ± 55	115 ± 79	104 ± 59	NS

Superscript letters within each row indicate significant differences among groups that are marked with different letters, according to the post hoc Fisher LSD test (*p* < 0.05).

**Table 2:** Plasma metal contents in total, high-molecular-mass (HMM), and low-molecular-mass (LMM) fractions. Results are expressed as mean ± standard deviation (µg/L). ND, nondetected; NS, nonsignificant.

		Early Peak	Middle Peak	Late Peak	*p*-Value
Arsenic	Total	0.78 ± 0.72	0.81 ± 0.83	0.68 ± 0.50	NS
	HMM	0.17 ± 0.12 ^a^	0.16 ± 0.16 ^a^	0.077 ± 0.037 ^b^	0.0054
	LMM	0.71 ± 0.50	0.62 ± 0.63	0.63 ± 0.52	NS
Cadmium	Total	0.0024 ± 0.0016	0.0028 ± 0.0032	0.0033 ± 0.0034	NS
	HMM	0.0024 ± 0.0017	0.0027 ± 0.0031	0.0033 ± 0.0035	NS
	LMM	ND	ND	ND	-
Chromium	Total	6.3 ± 2.7	6.4 ± 2.9	5.9 ± 3.4	NS
	HMM	5.1 ± 2.2 a	5.1 ± 2.3 a	4.8 ± 2.8 b	0.049
	LMM	1.2 ± 1.2	1.0 ± 0.7	0.78 ± 0.84	NS
Cobalt	Total	1.5 ± 0.6	1.4 ± 0.4	1.4 ± 0.3	NS
	HMM	1.2 ± 0.5 ^a^	1.0 ± 0.5 ^a^	0.79 ± 0.14 ^b^	0.0054
	LMM	0.30 ± 0.072	0.29 ± 0.056	0.31 ± 0.050	NS
Copper	Total	1367.0 ± 283.6	1390.2 ± 173.5	1305.2 ± 217.1	NS
	HMM	1246.9 ± 206.8	1262.7 ± 173.5	1210.8 ± 154.4	NS
	LMM	23.8 ± 19.7	18.9 ± 14.3	19.6 ± 16.6	NS
Iron	Total	668.6 ± 346.8	673.7 ± 260.8	650.8 ± 194.6	NS
	HMM	687.1 ± 255.1	705.4 ± 212.5	678.5 ± 149.4	NS
	LMM	27.2 ± 16.0	22.4 ± 6.9	23.5 ± 7.9	NS
Lead	Total	0.025 ± 0.0062	0.024 ± 0.0062	0.021 ± 0.0034	NS
	HMM	0.025 ± 0.0064 ^a^	0.024 ± 0.0061 ^a^	0.021 ± 0.0029 ^b^	0.015
	LMM	ND	ND	ND	-
Manganese	Total	4.1 ± 3.7	4.2 ± 2.2	4.2 ± 5.3	NS
	HMM	4.1 ± 0.8	4.2 ± 0.7	4.0 ± 0.9	NS
	LMM	0.22 ± 0.18	0.23 ± 0.11	0.21 ± 0.40	NS
Molybdenum	Total	2.6 ± 0.7	2.7 ± 0.6	2.4 ± 0.7	NS
	HMM	2.7 ± 1.1	2.8 ± 0.9	2.9 ± 0.9	NS
	LMM	ND	ND	ND	-
Selenium	Total	120.3 ± 18.5	125.8 ± 20.5	123.0 ± 20.1	NS
	HMM	123.4 ± 17.5	117.2 ± 19.6	111.3 ± 16.3	NS
	LMM	2.2 ± 1.9	1.7 ± 0.9	1.8 ± 1.7	NS
Zinc	Total	725.3 ± 154.3	757.0 ± 126.9	736.7 ± 126.9	NS
	HMM	731.5 ± 233.7	727.8 ± 191.2	723.3 ± 282.4	NS
	LMM	36.4 ± 15.8	39.2 ± 14.7	35.8 ± 24.1	NS

Superscript letters within each row indicate significant differences among groups that are marked with different letters, according to the post hoc Fisher LSD test (*p* < 0.05).

## Data Availability

The data that support the findings of this study are available on request from the corresponding author.
